# Location and neutrality

**DOI:** 10.1007/s11098-026-02604-z

**Published:** 2026-09-06

**Authors:** Alberto Carrascon

**Affiliations:** University of St Andrews, St Andrews, UK

**Keywords:** Location, Neutrality, Functionality, Exactness

## Abstract

I characterize two different senses in which a formal theory of location can be said to be neutral: neutrality with respect to some locational principles, and neutrality with respect to some controversial cases that have been presented as counterexamples to some principles. Theories neutral in the second sense are often weaker than theories neutral in the first. To illustrate this, I consider some popular principles and some of the counterexamples that have been used to argue against them in the literature, including inexact objects, cases of Nested Multilocation, and failures of Additivity. I then show that no currently existing theory of location built on just one primitive is able to account for all the cases that I have discussed. Lastly, I present a theory of location based on just one primitive which can account for all the discussed cases.

## Introduction

This paper is about neutrality in formal theories of location. By neutrality I mean the ability to remain impartial with respect to some controversial principles or scenarios, while also characterizing location in a way that aligns with our common intuitions on the matter (that is, while entailing those principles that are uncontroversial and virtually universally accepted).^1^ In other words, a perfectly neutral theory of location would be the weakest theory that still captures all the “analytic” principles regarding location. Such a theory would then be a very useful tool, because it would provide us with an ideal common ground from which to evaluate its competing expansions.

Of course, it is not easy to decide which principles should be included and which should not. One cannot simply consider all matters of controversy in the literature as automatically excluded from a neutral theory, and everything else as analytic: the counterexamples that have been proposed against a certain principle might be misguided, and, conversely, the mere fact that nobody has produced a counterexample to a certain principle is by no means an indicator that that principle should be uncritically accepted. Moreover, there is a prior difficulty that has to do with the relation between principles and counterexamples. It is often the case that a counterexample contains more assumptions than those which would be strictly necessary to refute the principle it is directed against, and, as a result, even if a theory is compatible with the negation of the principle, it may be unable to account for the scenario presented in the counterexample. We can thus distinguish two ways of being neutral: one is impartiality with respect to controversial principles, and the other consists in the ability to account for scenarios that have been presented as counterexamples. These two can come apart more easily than one may think. To illustrate this, in the first section of this paper I will consider some scenarios that have been extensively discussed in the literature as counterexamples to Exactness (§ 1.1) and to Functionality (§ 1.2). In the second section I will then propose a new theory of location that is built on just one primitive, exact location, and is able to account for all the cases that I will have discussed up to that point (as I will show, such a theory has not yet been developed). I will present the definitions for the theory, and show how they allow to account for the discussed counterexamples, in § 2.1, and reserve discussion of the axioms for § 2.2.

Since I will discuss principles and counterexamples before presenting a rigorous theory of location, I will need to talk about the various locational relations that are most commonly discussed before I have the chance to provide rigorous definitions for them. For this reason, I will now provide at least some intuitive characterizations of the notions in question. I will focus on the four most discussed relations of location: exact location, weak location, entire location, and pervasive location.

*Exact location* (@): an object is exactly located at a region when the object is in the region and the two have the same shape and size. An exact location is, in Parsons’ (2007) words, the “shadow in substantival space” of its occupant.

*Weak location* (@_w_): an object is weakly located at a region when the region is not completely free of the object. This is the most generic sense in which an object can be located somewhere; for example, I count as weakly located at my home even when I’m just sticking my hand in through the door, and the rest of my body is still outside. Since it is so generic, weak location is supposed to be entailed by all the other sorts of location: if I am located somewhere in any way, I also count as being weakly located there.

*Entire location* (@_e_): An object is entirely located at a region when all of the object lies within the region (the region may, but need not, be larger than the object). This is one of the most familiar senses in which we say that something is somewhere; examples include me being in my office, my office being in a university building, and so on. (Notice that the second *relata* in these sentences should be taken as regions, not as occupants).

*Pervasive location* (@_p_): an object is pervasively located at a region when all of the region is filled by the object (the object may, but need not, be larger than the region). This is in a sense the dual of entire location; for example, I am pervasively located at (or, more succinctly: I pervade) the region at which my right hand is exactly located, my right hand pervades the region at which my right thumb is exactly located, and so on.

## Neutrality

The notion of neutrality in relation to theories of location can be characterized in at least two different ways: neutrality with respect to controversial principles, and neutrality with respect to specific counterexamples. A theory is neutral in the first sense if, given a certain locational principle P, the theory is compatible both with P and with its negation (in other words, the theory could be extended by adopting P as an axiom without inconsistency, and the same goes for not-P). A theory is neutral in the second sense if, given a certain counterexample C, the theory can account for C, i.e. it has some models that can be taken to adequately represent C.^2^ I take a counterexample to be something like the description of a scenario that one considers to be a genuine metaphysical possibility. Counterexamples are generally aimed at specific principles in such a way that, if a theory can account for counterexample C to principle P, then it is compatible with the negation of P. However, it is not always the case that, if a theory is compatible with the negation of P, then it can account for all the counterexamples to P. This is because counterexamples sometimes involve specific details that might be ruled out by the theory in some other way. To illustrate this, I will consider some popular counterexamples to two principles which, since Parsons (2007), have been the focus of much discussion in the literature: Exactness and Functionality.

### Exactness

Exactness states that any object that has a weak location has an exact location.^3^

$$\:\mathrm{E}\mathrm{X}.\:\exists\:\mathrm{y}(\mathrm{x}@_\mathrm{w}\mathrm{y})\:\to\:\:\exists\:\mathrm{z}(\mathrm{x}@\mathrm{z})$$

Since weak location is supposed to be the weakest form of location, one that every located object exemplifies, the principle effectively states that, if an object is located at all, then it has an exact location. Two of the most discussed counterexamples to Exactness, both presented by Parsons (2007), involve a point-like object in gunky space and an omnipresent object in junky space.

Gunky space comprises only composite, extended regions, while a point-like object is unextended and mereologically simple. It is, in Parson’s words, “more spatially fine-grained than space itself”; as a result, the point-like object is weakly and entirely located at an infinite series of smaller and smaller regions, but – at least intuitively – there is no region that counts as its exact location (and also no region that counts as being pervaded by it).^4^ For this reason, this case is not only a counterexample to Exactness, but also to a similar principle, which may be dubbed E-Exactness, stating that if an object is entirely located then it is exactly located.

$$\:\mathrm{E}-\mathrm{E}\mathrm{X}.\:\exists\:\mathrm{y}(\mathrm{x}@_\mathrm{e}\mathrm{y})\:\to\:\:\exists\:\mathrm{z}(\mathrm{x}@\mathrm{z})$$

This principle is entailed by regular Exactness under the commonly accepted assumption that entire location entails weak location. Of course, the converse implication does not hold. To account for point-like objects in gunky space, a theory must thus be compatible not only with the negation of Exactness, but also with the negation of E-Exactness. A theory could be neutral with respect to Exactness, but still unable to account for point-like objects in gunky space, if it ruled them out but allowed for some other type of inexact object. For example, consider a theory that takes pervasion as a primitive, defines “x is exactly located at y” as “x pervades y and any region pervaded by x is a part of y”, and defines “x is entirely located at y” as “x is exactly located at a subregion of y”. Such a theory would entail E-Exactness, because, given the definition of entire location, it would require that any object that is entirely located at a region y is also exactly located at a mereological extension of y. However, it would not entail Exactness, because it would still allow for objects that are pervasively (and thus, we might assume, also weakly) but not exactly located (for example, an object such that no pervasive location of it includes as parts all other pervasive locations of it).

The case of an omnipresent object in junky space is in a sense the dual of the previous one. A space is junky if it has no maximal region, or, in other words, if any region of it is a proper part of another region of it: just like gunky space has no *minima*, junky space has no *maxima*. An omnipresent object is one that is weakly located at every region of the space. Given the junkiness of the space in question, the omnipresent object will also intuitively pervade every region of it, but it will have no exact or entire location. Therefore, this case is a counterexample not only to Exactness, but also to the principle we may call “P-Exactness”, which states that, if an object has a pervasive location, then it has an exact location.

$$\:\:\mathrm{P}-\mathrm{E}\mathrm{X}.\:\exists\:\mathrm{y}(\mathrm{x}@_\mathrm{p}\mathrm{y})\:\to\:\:\exists\:\mathrm{z}(\mathrm{x}@\mathrm{z})$$

Similarly to the case of E-Exactness, Exactness entails P-Exactness under the virtually universal assumption that pervasive location entails weak location. P-Exactness, on the other hand, does not entail Exactness, as the case of the point-like object in gunky space can be taken to show (in that scenario P-Exactness could hold even though Exactness is violated). So, a theory that accounts for omnipresent objects in junky space must be compatible not only with the negation of Exactness, but also with the negation of P-Exactness, and, of course, with the possibility of junky spaces.

### Functionality

Functionality is the principle stating that any object can have at most one exact location. More precisely, it says that if an object x is located at regions y and z, then y and z are identical.

$$\:\mathrm{F}\mathrm{U}\mathrm{N}.\:(\mathrm{x}@\mathrm{y}\:\wedge\:\:\mathrm{x}@\mathrm{z})\:\to\:\:\mathrm{y}\hspace{0.17em}=\hspace{0.17em}\mathrm{z}$$

One of the most discussed cases that have been used to argue against Functionality involves enduring objects, which “persists by being wholly present at more than one time” (Lewis, 1986: 202). If we interpret endurance as having multiple exact locations at multiple times, any enduring object constitutes a counterexample to Functionality.^5^ Under these accounts of endurance, another common assumption is that enduring objects are not, in addition, also exactly located at the temporally extended regions composed by some of their instantaneous exact locations. After all, enduring objects are three-dimensional, and thus, one may think, they can only be exactly located at temporally unextended, three-dimensional regions. If this is true, enduring objects also serve as counterexamples to Additivity, the principle stating that any object is exactly located at all the mereological fusions of its exact locations.^6^

$$\:\:\:\mathrm{A}\mathrm{D}\mathrm{D}.\:(\mathrm{x}\::\:\mathrm{x}\mathrm{x}\:\to\:\:\mathrm{y}@\mathrm{x})\:\to\:\:(\mathrm{F}\mathrm{x}\mathrm{x}\mathrm{z}\:\to\:\:\mathrm{y}@\mathrm{z})$$

Supporters of multilocation usually reject Additivity (see for example Gilmore, 2007, Eagle, 2010); however, there are also theories that include a non-functional but additive version of exact location (for example, Eagle, 2016). Such theories are neutral with respect to Functionality, but still unable to account for cases involving enduring objects.

Another popular counterexample to Functionality involves immanent universals, which are usually taken to be located wherever and whenever their instances are (Armstrong, 1978; Bigelow, 1988).^7^ Immanent universals can give rise not only to cases of multilocation *simpliciter*, but also to cases of *nested* multilocation, in which one location of an object x is the proper part of another location of x (Costa & Calosi, 2022). This is because one and the same universal is often instantiated by multiple mereologically commensurable objects. For example, if a composite whole is red all over, many of its proper parts are also red, which plausibly means that the universal redness is exactly located both at the location of the whole and at the locations of its red parts (and, as is generally assumed, the locations of the parts are themselves parts of the location of the whole).

If one wanted to rule out nested multilocation without ruling out multilocation more broadly, one could resort to a principle like the following, stating that if an object x is located at two regions y and z, neither region is a proper part of the other.

$$\:\mathrm{N}\mathrm{O}\:\mathrm{N}.\mathrm{M}.\:(\mathrm{x}@\mathrm{y}\:\wedge\:\:\mathrm{x}@\mathrm{z})\:\to\:\:\neg\:(\mathrm{P}\mathrm{P}\mathrm{y}\mathrm{z}\:\vee\:\:\mathrm{P}\mathrm{P}\mathrm{z}\mathrm{y})$$

No Nested Multilocation is entailed by Functionality, while the converse entailment does not hold. No Nested Multilocation, moreover, rules out non-trivial cases of Additivity: if nothing can be located at distinct regions one of which is part of the other, no multilocated object can also be located at the fusions of its locations. No Nested Multilocation is similar to a slightly stronger principle ruling out all multilocated objects that have overlapping distinct locations, which has been proposed as a possible axiom by Correia (2022: 572) under the name Restricted Multilocation. In any case, most theories on the market (even among those that allow for multilocation) do not explicitly reject nested multilocation: it is just ruled out by their definition of exact location. This is the case, for example, for Eagle (2010), Loss (2021), and Correia (2022). As a result, these theories cannot accommodate for many scenarios involving immanent universals, despite being neutral with respect to Functionality.

Many cases involving immanent universals also violate Additivity. For example, the immanent universal “being a cat” is located at all the regions where a cat is located; it is not, however, located at the fusion of all those regions, because the fusion of all cats is not itself a cat (assuming, of course, that more than one cat exists). More examples of this sort can be construed with universals like “being small”, or “being self-connected”. Therefore, to account for many cases involving immanent universals, a theory must also be compatible with the negation of Additivity.

If we assume, as seems plausible, that immanent universals are mereologically simple, many of them also turn out to be extended simples. Extended simples are commonly taken to be objects that have no proper parts, but are exactly located at a mereologically complex region (Pickup, 2016; Eagle, 2019). Under this mereological understanding of extension, all extended simples are spanners (even though not all spanners are extended simples). An object x is a spanner iff it is exactly located at a composite region r, but none of the parts of x are exactly located at any of the proper subregions of r (Hudson, 2005: 101). Therefore, to account for many cases involving immanent universals, a theory should also be able to account for extended simples (and spanners) in addition to being neutral with respect to Functionality.

Notice that, as pointed out by Calosi (2023), the mereological one is not the only available account of extension, nor, arguably, the best. The mereological account of extension assumes that all composite regions are extended, and all simple regions are unextended, but this cannot be taken to hold for all spaces unless explicitly postulated.^8^ Extended simple regions can only be ruled out through preliminary assumptions regarding the admissible structures of spaces and the geometrical or topological properties of regions, which are not expressible in purely locational terms. Furthermore, there could be regions that are composite but unextended: for example, a point-like region composed of two distinct but co-located point-like regions. A simple object occupying such a region should not intuitively count as an extended simple, even though its location is composite. In this case, as we will see in the following section, there is a way to rule this out in purely locational terms. But anyways, unless we have done so we cannot rest assured that an object is extended just because its location is composite.

The distinction between spanners and extended simples is significant, but often overlooked in the literature. For example, Correia (2022) seems to suggest that Eagle’s (2010) theory is not *neutral* (in his technical, defined sense of *neutrality*, which consists in being compatible with violations of Functionality, Exactness, and No Spanners^9^) because it cannot account for extended simples. However, Eagle’s theory does allow for the possibility of spanners that are not extended simples, and so it is, strictly speaking, *neutral* in the sense of being compatible with some violations of Functionality, Exactness, and No Spanners (one could say that it follows the letter, but not the spirit, of Correia’s definition of neutrality). This example illustrates the difference between a notion of neutrality defined on general principles and a notion of neutrality based on the ability to account for specific cases, and shows how a theory that is compatible with the negation of a certain principle can fail to account for all the cases that have been proposed as counterexamples to the principle in question.

## A theory of location

Currently, no theory of location built on just one primitive can account for all the cases that have been considered in the previous section. To my knowledge, the only existing theory that can account for all the discussed scenarios is the one proposed by Correia (2025), but this theory needs at least two primitives to be formulated (in addition to those required by its logical and mereological framework): the first is a locational relation (one that Correia calls “direct weak location”), and the second is the relation of “being a proxy for”. The theory stipulates that objects can be multiply located by having multiple “proxies”, whose locations they inherit, and posits a distinction between being located directly and indirectly (that is, via a proxy). For example, an enduring object *e* can be located at both *t1* and *t2* by having one proxy (maybe an event involving *e*, as suggested by Correia) located at *t1*, and another at *t2*. This yields the ability to account for many controversial cases in the literature, but it does so at the cost of reduced ideological parsimony.

It should be noted that, in general, accounting for many controversial cases becomes way easier as soon as we help ourselves to a second primitive: for example, a theory which has both exact and weak location as primitives can easily account for cases involving nested multilocation, non-additive multilocation, and spanners, simply by having primitive exact location, and can also easily avoid the entailment of Exactness and related principles by having primitive weak location. The argument usually adduced in the literature against theories with two locational primitives is that they would posit many brute necessary facts regarding location (see e.g. Leonard, 2014, Eagle, 2019). For example, if both exact location and weak location were primitive, the fact that any object is weakly located wherever it is exactly located would be one such unexplained necessity. I do not find this argument very convincing: the necessary facts involving the two primitives of the theory don’t strike me as particularly unappealing, as long as they are all entailed by the axioms of the theory. Instead of expecting our theory of location to explain why exact location entails weak location via the definition of one of the two, we could just take this fact as axiomatic, that is, as constitutive of the meanings of both weak and exact location.

In any case, the argument above only applies to theories with two locational primitives, which is why the theory in Correia (2025) is immune from it, as long as the proxy relation is not taken to be a locational primitive.^10^ Still, on grounds of ideological parsimony alone, we have good reasons to prefer a theory built on one primitive to a theory built on two primitives (in addition to the logical and mereological ones), as long as the two are more or less equally able to account for the relevant scenarios. But, as already mentioned, the one-primitive theories in the literature don’t fare nearly as well against the controversial cases we have been considering.

In fact, most of the theories that feature a non-functional version of exact location are still incompatible with cases of nested multilocation (this is the case for Eagle, 2010, Loss, 2021, and Correia, 2022). One theory that allows for nested multilocation can be found in Eagle (2016); this theory, however, has exact location as a primitive and Exactness as a theorem, meaning that it cannot account for inexact objects. A theory that is compatible both with nested multilocation and with inexact objects is the one in Eagle (2019), which takes weak location as a primitive and defines from it both a functional and a non-functional version of exact location. The non-functional version of exact location, however, turns out to be additive, which means that all multiply located objects are also located at the fusions of their locations. As seen in the previous section, some accounts of enduring objects, as well as some cases involving immanent universals, are incompatible with the assumption of Additivity, which means that this theory cannot account for them. And so, as far as I’m aware, no existing theory with just one locational primitive is compatible with cases of nested multilocation, failures of the Exactness principles, and violations of Additivity, taken together.

There is a widespread belief in the literature that any theory that has exact location as a primitive cannot be compatible with failures of Exactness (and related principles). For example, Correia (2022: 563) argues that no theory compatible with the negation of Exactness can define weak location in terms of exact location because of cases like the point-like object in gunky space. The argument rests on the fact that one can construct, based on such cases, two models that differ with respect to weak location (because the point-like object is weakly located at different regions depending on the model) but not (allegedly) with respect to exact location (because the point-like object is never exactly located anywhere). Eagle (2019: 182-3) offers a very similar argument, based on the alleged possibility of scenarios that “agree in all the facts about exact location but disagree on weak location – e.g., they agree that x has no exact location, but disagree on where x is weakly located”. But later in this section we will see how, contrary to this widespread belief, there are ways to define the locational relations with exact location as a primitive without entailing the Exactness principles.

In fact, the theory that I will present in this section takes exact location as a primitive, and it is compatible with all the cases considered in the previous section. I will formulate the theory in a one-sorted language of classical first-order logic with identity, meaning that I will use the same variables for regions and occupants, as I have been doing up to this point. Of course, the notions of region and occupant can still be easily defined in the theory: a region is something such that something is weakly located at it, and an occupant is something such that it is weakly located somewhere and it is not a region (the relevance of the latter conjunct will become clear shortly). I will first present the definitions of the locational relations based on exact location and show how they make the theory compatible with the previously considered cases, and then I will discuss the axioms of the theory.

Before I start, let me point out that the theory I am about to present is in no way supposed to be perfectly neutral, or to entail all and only analytic truths regarding location. The theory is designed to account for a specific set of cases, those that have been considered in the previous section, but it is always possible to construct more specific scenarios that the theory cannot account for. For example, the theory can account for omnipresent objects in junky space, but only if the omnipresent objects in question have some parts that are exactly located. For this reason, the theory would be unable to account for an atomic omnipresent object in junky space. Such a scenario can be relevant, since God, Who may be the best example of an omnipresent object, is plausibly mereologically simple. Similarly, the theory can account for point-like objects in gunky space, but might yield unwanted results in cases of multiple entire location, that is, cases where a point-like object is entirely located at multiple disjoint pluralities of regions. In what follows I will explain more accurately how such cases can be problematic for the present theory.

### Definitions

Let us start with entire location. The definition I will adopt is disjunctive: it says that an object x is entirely located at a region y iff x is exactly located at a subregion of y, or there is an object z such that x is a part of z and z is exactly located at y.

$$\:\mathrm{E}.\:\mathrm{L}\mathrm{O}\mathrm{C}.\:\mathrm{x}@_\mathrm{e}\mathrm{y}\hspace{0.17em}=\hspace{0.17em}_\mathrm{def}\:\exists\:\mathrm{z}(\mathrm{P}\mathrm{z}\mathrm{y}\:\wedge\:\:\mathrm{x}@\mathrm{z})\:\vee\:\:\exists\:\mathrm{w}(\mathrm{P}\mathrm{x}\mathrm{w}\:\wedge\:\:\mathrm{w}@\mathrm{y})$$

The first disjunct is a familiar one: it says that an object is entirely located at a region when it is located at one of its subregions, the same as Parsons’ (2007) definition with exact location as a primitive. That definition requires any entirely located object to be exactly located, which is why it entails E-Exactness. The second disjunct is what allows my proposed definition to avoid the entailment of E-Exactness: it doesn’t require x to be exactly located anywhere, it just requires it to be part of something that is exactly located at y. This allows objects that are not exactly located to still count as entirely located at some region. For example, a point-like object p can be entirely located at a gunky region r without being exactly located at any subregion of r, as long as p is part of something that is exactly located at r.

This may seem rather underwhelming, since the fact that p counts as entirely located at r is dependent on the existence of an extension of p that is located at r. However, the definition becomes more promising under the assumption of the principles of (i) Conditional Reflexivity, (ii) Unrestricted Binary Fusion, and (iii) Locational Supplementation, which I will assume as axioms of the present theory. These principles state the following: (i) any region is exactly located at itself; (ii) binary mereological fusion is unrestricted; and (iii) if some part of x is exactly located at y and no part of x is weakly located at a region disjoint from y, then x is exactly located at y. These principles ensure that: (i) there is something that is located at r (namely, r itself); (ii) the fusion of p and r exists; and (iii) the fusion of p and r is also located at r (because a part of it, namely r, is exactly located at r, and no part of it is weakly located at a region disjoint from r).

This is how the definition allows us to deal with cases of point-like objects in gunky space. Imagine our point-like object, p, is somewhere in a gunky space. Given the gunkyness of the space, p does not have an exact location, yet it should intuitively count as weakly and entirely located in an infinite series of nested regions. Take one of these regions, r. By Conditional Reflexivity, we know that r is located at itself, and by Unrestricted Binary Fusion, we know that p and r have a fusion (call it f). By Locational Supplementation, we know that f is exactly located at r. Since p is a part of f, we know that p is part of something that is located at r (namely, f), and thus, by the second conjunct of the definition of entire location, p counts as being entirely located at r. Later we will also see how, given the definition of weak location, p counts as being weakly located at all the regions where it is entirely located, as one would expect.

**Fig. 1:**
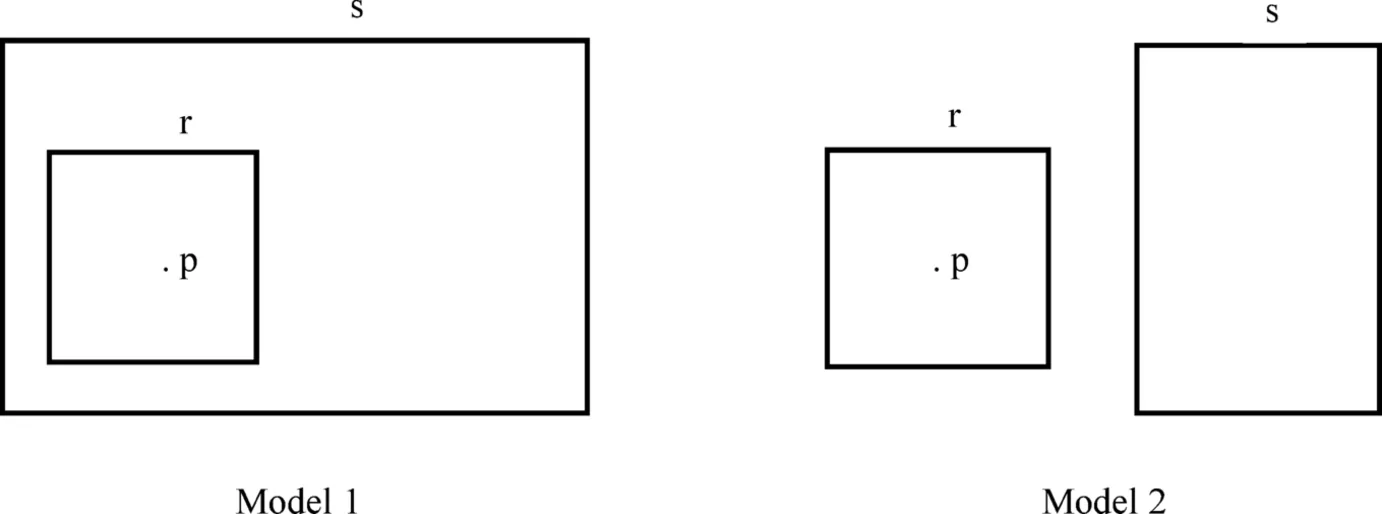
Two models of a point-like object in gunky space

Notice how, given these assumptions, it is impossible that two scenarios differ with respect to entire location but not with respect to exact location. Consider the two models in the figure below, where a point-like object p is located somewhere in gunky space. In the first model, p is entirely located at both r and s, while in the second model it is entirely located at r but not at s. In both models, p lacks an exact location, but still the two models differ with respect to exact location: in the first model, the fusion of p and s is exactly located at s, while in the second model, due to Locational Supplementation, the fusion of p and s is not exactly located at s, given that, although one of its parts is exactly located at s, it does also have a part (namely, p) that is weakly located at a region disjoint from s (that is, r). This shows how, given our assumptions, any two scenarios that differ with respect to entire location must also differ with respect to the exact location of some of the entities they include, and thus illustrates how the present theory can account for some cases involving inexact objects even though it has only exact location as a primitive (Fig. 1).

As previously mentioned, one scenario that can spell trouble for the present theory involves a point-like object in gunky space that is entirely located at multiple disjoint pluralities of regions. According to the definition of entire location, an object p counts as entirely located at a region r if a mereological extension of it (which can be the fusion of p and r) is exactly located at r. Given Locational Supplementation, the fusion of p and r is exactly located at r if no part of it is weakly located at a region disjoint from r. But if p is entirely and weakly located at multiple disjoint pluralities of regions, it can be the case that it is weakly located also at a region disjoint from r, and so the theory fails to entail that the fusion of p and r is exactly located at r.^11^ I believe such shortcomings could be avoided by adopting an improved version of Locational Supplementation, one better equipped to deal with cases of inexactly located objects that have multiple disjoint entire locations; however, I have not been able to find a satisfying alternative to this principle.

The definition of pervasive location is disjunctive as well. It says that an object x pervades a region y iff there is something that is a part of x and is exactly located at y, or x is exactly located at a region that includes y as a part.

$$\:\mathrm{P}.\:\mathrm{L}\mathrm{O}\mathrm{C}.\:\mathrm{x}@_\mathrm{p}\mathrm{y}\hspace{0.17em}=\hspace{0.17em}_\mathrm{def}\:\exists\:\mathrm{z}(\mathrm{P}\mathrm{z}\mathrm{x}\:\wedge\:\:\mathrm{z}@\mathrm{y})\:\vee\:\:\exists\:\mathrm{w}(\mathrm{P}\mathrm{y}\mathrm{w}\:\wedge\:\:\mathrm{x}@_\mathrm{w})$$

In other words, x can pervade y either by having a part that is located there, or by being located at a mereological extension of y (or both). The first disjunct is there to avoid the entailment of P-Exactness and account for pervading objects that are not exactly located anywhere (as long as some part of them is exactly located somewhere). For example, it can be used to say that an omnipresent object pervades every region of a junky space, even though it is not exactly located at any of them. The second disjunct, on the other hand, allows objects that have no exactly located proper parts to pervade some regions (as long as they are exactly located somewhere). For example, it allows spanners to pervade all the proper subregions of their composite locations, even though no parts of the spanners are exactly located at any of the subregions. This disjunctive definition makes it so that the theory can accommodate both cases involving spanners (and extended simples) and cases of inexactness (but only as long as the inexact objects in question have some exact parts, which, as previously mentioned, represents a limitation of the present theory).

Last is the definition of weak location. An object x is weakly located at a region y iff there is some part of x that is entirely or pervasively located at y.

$$\:\mathrm{W}.\:\mathrm{L}\mathrm{O}\mathrm{C}.\:\mathrm{x}@_\mathrm{w}\mathrm{y}\hspace{0.17em}=\hspace{0.17em}_\mathrm{def}\:\exists\:\mathrm{z}(\mathrm{P}\mathrm{z}\mathrm{x}\:\wedge\:\:(\mathrm{z}@_\mathrm{e}\mathrm{y}\:\vee\:\:\mathrm{z}@_\mathrm{p}\mathrm{y}\left)\right)$$

This ensures that any object that is entirely or pervasively located somewhere is also weakly located there, because of the reflexivity of parthood. Consequently, an object can be weakly located without being exactly located, as long as some part of it is entirely or pervasively located; and so Exactness is not entailed.

It is interesting to consider which entailments hold between the different locational relations so defined. As we have seen, given the definition of weak location, an object that is entirely located somewhere is also weakly located there, and similarly an object that is pervasively located somewhere is also weakly located there. In addition, given the definitions of entire and pervasive location, an object that is exactly located somewhere also counts as being both entirely and pervasively located there. And so it follows that if an object is exactly located somewhere it is also weakly located there, which means that weak location is entailed by all the other locational relations. This is good, because I take it to be part of the intuitive meaning of weak location that it is the most general form of location.

All the implications in the opposite direction, on the other hand, do not follow. The fact that an object is weakly located does not entail that the object is exactly, entirely, or pervasively located anywhere, and the fact that an object is entirely or pervasively located does not entail that the object is exactly located anywhere. As we have seen, this allows our theory to be compatible with the negation of any Exactness-related principle. Another feature of the theory is this: the fact that an object is both entirely and pervasively located at a certain region does not entail that the object is exactly located at that region. This is a difference with respect to stronger theories, like Parsons (2007), where the conjunction of entire and pervasive location entails exact location. I take this to be a positive feature of the theory, but some may disagree. Some may argue that if an object is both entirely and pervasively located at a region it should also count as being exactly located at that region; this seems reasonable enough, if we are considering cases of functional exact location, or even of multiple disjoint exact locations. However, I believe that it should not hold in a theory that allows for nested multilocation.

**Fig. 2:**
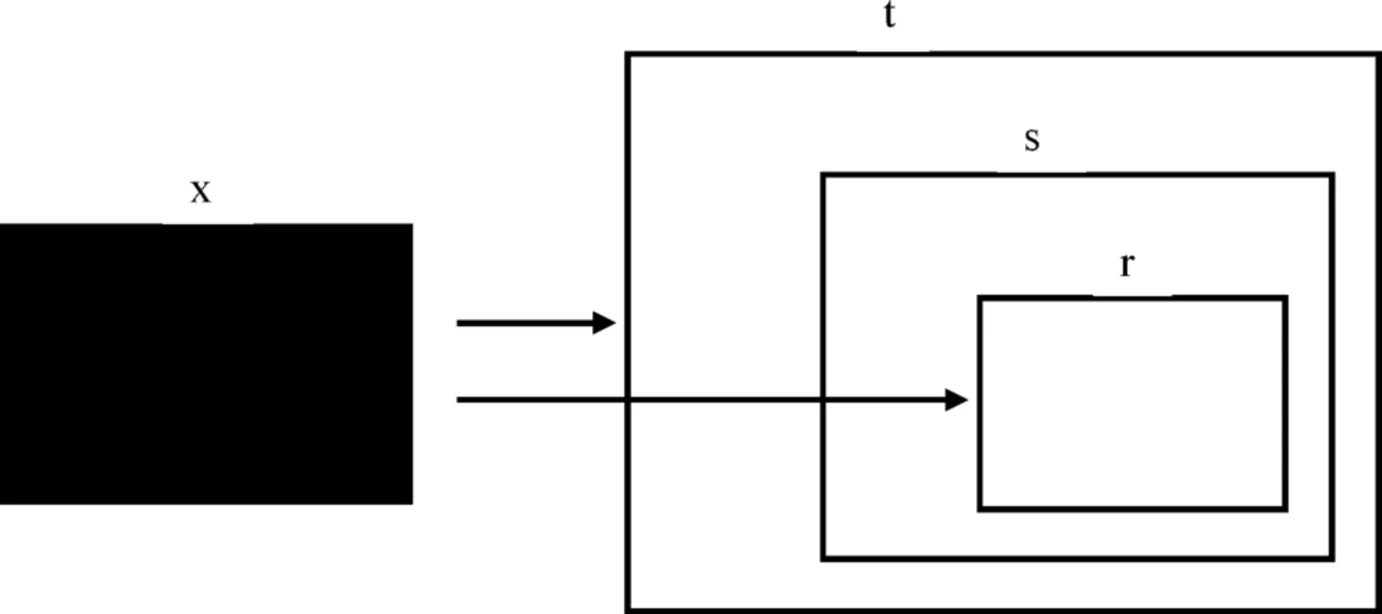
Nested multilocation (the arrows represent exact location)

To see this, consider the case illustrated in the figure below, in which three regions, r, s, and t, are such that r is a proper part of s and s is a proper part of t. Suppose that an object x is exactly located both at r and at t, but not at s. However, according to our definitions, x both pervades s (because s is a proper part of an exact location of x) and is entirely located at s (because it is located at a subregion of s). If this scenario is admissible, the conjunction of entire and pervasive location should not entail exact location (Fig. 2).

I have already shown how the proposed definitions allow the theory to be compatible with the negations of the Exactness principles, and how the definition of pervasion in particular allows the theory to accommodate cases in which a spanner is located at a certain region, and pervades the subregions of that region, without having any part that is exactly located at any of the subregions. The fact that a spanner (or a simple object) can be exactly located at a composite region depends, of course, on the fact that exact location is the primitive of our theory, and so we can construct models in which it is instantiated very liberally. This is also why the theory is compatible with cases that violate Functionality, including cases of nested multilocation, and why the theory is compatible both with cases that respect Additivity and with cases that violate it.

Exact location, however, should not be instantiated in a completely arbitrary fashion; we still need to restrict its instantiation in such a way that it aligns with how we commonly understand the concept. And, more generally, while we want our theory to be weak enough to accommodate a plethora of controversial cases, we also want it to be strong enough to entail those principles that are generally accepted as valid or even as constitutive of the meaning of expressions associated with the locational relations. This can be achieved by adding the desired principles as axioms.

### Axioms

The principles regarding location can be divided into two groups: first, the principles that concern the interactions of locational relations with each other and with the logical constants, including identity (the “pure” principles of location); and, second, the principles that concern the interactions of locational relations with parthood and other mereological relations (the “mereo-locational” principles). Before I delve into the locational axioms, I should say that I preliminarily assume the framework of Extensional Mereology (EM), which includes the usual ordering axioms for parthood (Reflexivity, Antisymmetry, Transitivity) as well as Strong Supplementation as a decomposition principle.^12^ This is a common and relatively uncontroversial move in the theory of location. In addition to the axioms of EM, I will also assume Unrestricted Binary Fusion as a composition principle, as I anticipated when discussing the definition of entire location. Recall that Unrestricted Binary Fusion, which states that any two things have a fusion, is needed to entail that, whenever some x is entirely located at a region r, there is something that is the fusion of x and r, which is crucial to ensure that the definition applies generally enough.

Some may wonder why I do not simply adopt Classical Extensional Mereology (CEM) as a framework, which would amount to assuming Unrestricted Fusion instead of Unrestricted Binary Fusion. Basically, adopting CEM would entail that all pluralities of objects (rather than just pairs of things) compose something. This turns out to make no difference in finite domains, since binary fusion can of course be iterated, but it makes a difference when the domain is infinite: in that case, Unrestricted Fusion entails that there is the fusion of all the objects of the domain, while Unrestricted Binary Fusion does not, because it only guarantees that pluralities of finitely many objects have a fusion. This is important because we want our theory to be able to account for cases involving junky spaces, that is, spaces without a maximal region, and, as we will see, we also want to adopt Preservation of Regionhood, an axiom to the effect that the fusion of some regions is a region. Unrestricted Fusion plus Preservation of Regionhood would entail that all spaces have a maximal region, while Unrestricted Binary Fusion allows for spaces with infinitely many regions to be junky, which is why I have preferred it in the context of the present theory.

Let us now proceed to the discussion of the pure principles of location. As we have seen, another assumption we need is that regions are exactly located at themselves. We can achieve this by adopting Conditional Reflexivity, which states that, if anything is weakly located at y, then y is located at itself.

$$\:\mathrm{C}\mathrm{O}\mathrm{N}.\:\mathrm{R}\mathrm{E}\mathrm{F}.\:\mathrm{x}@_\mathrm{w}\mathrm{y}\:\to\:\:\mathrm{y}@\mathrm{y}$$

Since, as previously mentioned, regions can be defined as things at which something is weakly located, this ensures that every region is self-located. Interestingly, the choice between having self-located regions and having regions that are not located anywhere has frequently been treated as one devoid of any relevant philosophical implications; for example, Parsons (2007: 224) states that he does “not see how to assign any metaphysical significance to this question”, and shortly after that Conditional Reflexivity and its denial are “equally good stipulations”. But, as we have seen, the choice of self-located regions has important consequences for the present theory, because it allows us to use exact location to define entire location (and, indirectly, weak location) without entailing Exactness or E-Exactness. This would not be possible if we assumed that regions are not located anywhere, because then there could be regions at which nothing is exactly located, and so not all entirely located objects would be part of something that is exactly located.

Conditional Reflexivity states that any region is exactly located at itself, but it does not rule out that some region x may also be exactly located at some other region y that is distinct from x. However, even though we want to allow for multilocated objects, I cannot see any reason why we may want to allow for multilocated regions. On the contrary, I believe multilocated regions should be ruled out. We can do so through an axiom of Conditional Functionality, stating that, if some x is weakly located at y, then any region at which y is exactly located is identical to y.

$$\:\mathrm{C}\mathrm{O}\mathrm{N}.\:\mathrm{F}\mathrm{U}\mathrm{N}.\:\mathrm{x}@_\mathrm{w}\mathrm{y}\:\to\:\:(\mathrm{y}@\mathrm{z}\:\to\:\:\mathrm{y}\hspace{0.17em}=\hspace{0.17em}\mathrm{z})$$

Conditional Functionality states that any region is exactly located at only one region, and that region is itself. Notice, however, that it does not entail Conditional Reflexivity, because, technically, Conditional Functionality is trivially satisfied in cases where regions are not exactly located anywhere, and so it is compatible with regions not having a location.

Conditional Functionality entails Antisymmetry, a theorem stating that, whenever x is located at y and y is located at x, x and y are identical.

$$\:\mathrm{A}\mathrm{N}\mathrm{T}.\:(\mathrm{x}@\mathrm{y}\:\wedge\:\:\mathrm{y}@\mathrm{x})\:\to\:\:\mathrm{x}\hspace{0.17em}=\hspace{0.17em}\mathrm{y}$$

Conditional Functionality also entails that two distinct regions cannot be co-located: for any regions x and y, if x and y are exactly located at the same region z, then x and y are identical (because they are identical to z, and identity is transitive). This, in turn, rules out cases in which a region is unextended but complex because it has many co-located unextended proper parts.

To be more precise, Conditional Functionality rules out unextended complex regions only under the plausible assumption that every part of a region is itself a region (because it only rules out co-located regions, but, if a region could have a part that is not a region, there could be an unextended region composed of co-located non-regions). To make this assumption explicit, it is convenient to define a predicate R that is true of all and only regions.

$$\:\: \mathrm{DEF}.\:\mathrm{R}\:\mathrm{R}\mathrm{x}\hspace{0.17em}=\hspace{0.17em}_\mathrm{def}\:\exists\:\mathrm{y}(\mathrm{y}@_\mathrm{w}\mathrm{x})$$

Then we can introduce the axiom of Dissectivity, stating that every part of a region is itself a region.^13^

$$\:\mathrm{D}\mathrm{I}\mathrm{S}\mathrm{S}.\:\mathrm{R}\mathrm{x}\:\to\:\:\left(\mathrm{P}\mathrm{y}\mathrm{x}\:\to\:\:\mathrm{R}\mathrm{y}\right)$$

As anticipated, we also want to adopt as an axiom Preservation of Regionhood, which states that all fusions of regions are regions.^14^ In this way, regionhood is closed both under parthood and under fusion.

$$\:\mathrm{P}\mathrm{R}.\:\mathrm{R}\mathrm{E}\mathrm{G}.\:(\mathrm{x}\::\:\mathrm{x}\mathrm{x}\:\to\:\mathrm{R}\mathrm{x})\:\to\:\left(\mathrm{F}\mathrm{x}\mathrm{x}\mathrm{y}\:\to\:\:\mathrm{R}\mathrm{y}\right)$$

Since they contain the parthood and fusion predicates, the last two axioms already count as mereo-locational principles, according to the classification we have previously sketched. We could here make a further distinction between mereo-locational principles that concern the mereological relations between regions, like Dissectivity and Preservation of Regionhood, and mereo-locational principles that concern the relations between the mereological structures of occupants and those of regions. The latter could be called “harmony” principles, following Uzquiano (2011).

One of the most popular principles of this sort is called Expansivity, and states that the locations of the parts of an object are themselves parts of the location of the object. More precisely, if x is a part of y, x is located at z, and y is located at w, then z is a part of w.^15^

$$\:\mathrm{E}\mathrm{X}\mathrm{P}.\:(\mathrm{P}\mathrm{x}\mathrm{y}\:\wedge\:\:\mathrm{x}@\mathrm{z}\:\wedge\:\:\mathrm{y}@_\mathrm{w})\:\to\:\:\mathrm{P}\mathrm{z}\mathrm{w}$$

In spite of its intuitive appeal, this principle must be rejected in the context of the present theory, because it is incompatible with the negation of Functionality. Expansivity says that *all* the locations of a certain part of y must be parts of *all* the locations of y; however, if exact location is not functional, there can be a model in which some part of y has two exact locations, *r* and *s*, and y itself has two locations, *t* and *u*, and *r* is part of *t* but not of *u*, and *s* is part of *u* but not of *t*. This violates Expansivity, but is compatible with the intuitive idea that motivates it: the idea that the parts of y must not extend further than y itself, or in other words, that any location of a part of y is the subregion of *some* location of y. It may thus seem that the right principle to capture this idea in the context of the present theory is one stating that, if x is a part of y and x is located at z, there is *some* w such that w is the location of y and z is part of w. I will call this principle *Non-Functional Expansivity.*^16^

$$\:\mathrm{N}.\mathrm{F}.\:\mathrm{E}\mathrm{X}\mathrm{P}.\:(\mathrm{P}\mathrm{x}\mathrm{y}\:\wedge\:\:\mathrm{x}@\mathrm{z})\:\to\:\:\exists\:\mathrm{w}(\mathrm{y}@_\mathrm{w}\:\wedge\:\:\mathrm{P}\mathrm{z}\mathrm{w})$$

Unfortunately, this is still not acceptable in the context of the present theory, because it is incompatible with the negation of Exactness. Non-Functional Expansivity says that, if x is part of y and located at z, then there is some region such that y is located there; but, if we do not accept the principle of Exactness, we cannot be sure that y has an exact location. This motivates us to adopt yet another principle, one that I will call Conditional Expansivity. According to Conditional Expansivity, if x is a part of y and x is located at z, then, *if y has an exact location*, then z is part of some exact location of y.

$$\:\mathrm{C}\mathrm{O}\mathrm{N}.\:\mathrm{E}\mathrm{X}\mathrm{P}.\:(\mathrm{P}\mathrm{x}\mathrm{y}\:\wedge\:\:\mathrm{x}@\mathrm{z})\:\to\:\:(\exists\:\mathrm{w}(\mathrm{y}@_\mathrm{w})\:\to\:\:\exists\:\mathrm{w}(\mathrm{y}@_\mathrm{w}\:\wedge\:\:\mathrm{P}\mathrm{z}\mathrm{w}\left)\right)$$

In other words, Conditional Expansivity says that the exact location of the part is a part of some exact location of the whole, as long as the whole has any exact location at all. I take this to capture the intuitive idea that motivates Expansivity while being compatible with the negations of both Functionality and Exactness.

Another relevant harmony principle is called Delegation, and says that, if a composite x is located at y, then for any subregion w of y there is some proper part of x that is exactly located at a region overlapping w.

$$\:\mathrm{D}\mathrm{E}\mathrm{L}.\:(\mathrm{x}@\mathrm{y}\:\wedge\:\:\exists\:\mathrm{z}(\mathrm{P}\mathrm{P}\mathrm{z}\mathrm{x}\left)\right)\:\to\:\:(\mathrm{P}\mathrm{w}\mathrm{y}\:\to\:\:\exists\:\mathrm{z}\exists\:\mathrm{v}(\mathrm{P}\mathrm{P}\mathrm{z}\mathrm{x}\:\wedge\:\:\mathrm{z}@\mathrm{v}\:\wedge\:\:\mathrm{O}\mathrm{z}\mathrm{w}\left)\right)$$

In spite of its plausibility, Delegation is not admissible in the present theory, because it is incompatible with the negation of Exactness. Delegation says that for any composite object x and any subregion w of its location there must be a proper part of x whose exact location overlaps w; however, if Exactness does not hold, nothing ensures that all the proper parts of x have an exact location, and so there could be some subregion of the location of x that is not overlapped by any exact location of a proper part of x. For example, if all the proper parts of x are point-like objects in a gunky space, no subregion of the location of x overlaps any exact location of any proper part of x (because the proper parts of x do not have any exact locations). This scenario violates Exactness, but it is still compatible with the intuitive idea that motivates Delegation as long as, for any subregion of the location of x, there is at least one proper part of x that is weakly located there.

While Expansivity is meant to say that the parts of x must not extend further than x itself, Delegation is meant to say that x must not extend further than its parts. In other words, the idea that motivates Delegation is that, if some region is not free of a composite object, then it is also not free of one of its proper parts. An expression of this idea that is compatible with the present theory is the following principle W-Delegation, which states that, if x is composite, then any weak location of x must also be a weak location of some proper part of x.^17^

$$\:\mathrm{W}-\mathrm{D}\mathrm{E}\mathrm{L}.\:\exists\:\mathrm{y}\left(\mathrm{P}\mathrm{P}\mathrm{y}\mathrm{x}\right)\:\to\:\:(\mathrm{x}@_\mathrm{w}\mathrm{z}\:\to\:\:\exists\:\mathrm{y}(\mathrm{P}\mathrm{P}\mathrm{y}\mathrm{x}\:\wedge\:\:\mathrm{y}@_\mathrm{w}\mathrm{z}\left)\right)$$

The last principle I will consider is also the last assumption needed to ensure that our definition of entire location can account for common cases of inexactness. The principle says that, if an object x is not located at a region y, then either no part of x is located at y or some part of x is weakly located at some region disjoint from y (or both). I will call this Locational Supplementation.

$$\:\mathrm{L}\mathrm{O}\mathrm{C}.\:\mathrm{S}\mathrm{U}\mathrm{P}.\:\neg\:\mathrm{x}@\mathrm{y}\:\to\:\:(\mathrm{P}\mathrm{z}\mathrm{x}\:\to\:\:\neg\:\mathrm{z}@\mathrm{y})\:\vee\:\:\exists\:\mathrm{w}\exists\:\mathrm{v}(\mathrm{P}\mathrm{w}\mathrm{x}\:\wedge\:\:\mathrm{D}\mathrm{v}\mathrm{y}\:\wedge\:\:\mathrm{w}@_\mathrm{w}\mathrm{v})$$

Essentially, this principle is meant to say that there are two ways in which x can fail to be exactly located at y: either none of its parts are exactly located at y, or, if one of its parts is exactly located there, there must be another part of it that is weakly located at a region disjoint from y.^18^

This concludes my discussion of the locational principles. Below I list all the definitions and axioms of the present theory:

$$\:\:\mathrm{E}.\:\mathrm{L}\mathrm{O}\mathrm{C}.\:\mathrm{x}@_\mathrm{e}\mathrm{y}\hspace{0.17em}=\hspace{0.17em}_\mathrm{def}\:\exists\:\mathrm{z}(\mathrm{P}\mathrm{z}\mathrm{y}\:\wedge\:\:\mathrm{x}@\mathrm{z})\:\vee\:\:\exists\:\mathrm{w}(\mathrm{P}\mathrm{x}\mathrm{w}\:\wedge\:\:\mathrm{w}@\mathrm{y})$$

$$\:\:\mathrm{P}.\:\mathrm{L}\mathrm{O}\mathrm{C}.\:\mathrm{x}@_\mathrm{p}\mathrm{y}\hspace{0.17em}=\hspace{0.17em}_\mathrm{def}\:\exists\:\mathrm{z}(\mathrm{P}\mathrm{z}\mathrm{x}\:\wedge\:\:\mathrm{z}@\mathrm{y})\:\vee\:\:\exists\:\mathrm{w}(\mathrm{P}\mathrm{y}\mathrm{w}\:\wedge\:\:\mathrm{x}@_\mathrm{w})$$

$$\:\:\mathrm{W}.\:\mathrm{L}\mathrm{O}\mathrm{C}.\:\mathrm{x}@_\mathrm{w}\mathrm{y}\hspace{0.17em}=\hspace{0.17em}_\mathrm{def}\:\exists\:\mathrm{z}(\mathrm{P}\mathrm{z}\mathrm{x}\:\wedge\:\:(\mathrm{z}@_\mathrm{e}\mathrm{y}\:\vee\:\:\mathrm{z}@_\mathrm{p}\mathrm{y}\left)\right)$$

$$\:\:\mathrm{DEF}.\:\mathrm{R}\:\mathrm{R}\mathrm{x}\hspace{0.17em}=\hspace{0.17em}_\mathrm{def}\:\exists\:\mathrm{y}\left(\mathrm{y}@_\mathrm{w}\mathrm{x}\right)$$

$$\:\:\mathrm{C}\mathrm{O}\mathrm{N}.\:\mathrm{R}\mathrm{E}\mathrm{F}.\:\mathrm{x}@_\mathrm{w}\mathrm{y}\:\to\:\:\mathrm{y}@\mathrm{y}$$

$$\:\:\mathrm{C}\mathrm{O}\mathrm{N}.\:\mathrm{F}\mathrm{U}\mathrm{N}.\:\mathrm{x}@_\mathrm{w}\mathrm{y}\:\to\:\:(\mathrm{y}@\mathrm{z}\:\to\:\:\mathrm{y}\hspace{0.17em}=\hspace{0.17em}\mathrm{z})$$

$$\:\:\mathrm{D}\mathrm{I}\mathrm{S}\mathrm{S}.\:\mathrm{R}\mathrm{x}\:\to\:\:(\mathrm{P}\mathrm{y}\mathrm{x}\:\to\:\:\mathrm{R}\mathrm{y})$$

$$\:\mathrm{P}\mathrm{R}.\:\mathrm{R}\mathrm{E}\mathrm{G}.\:(\mathrm{x}\::\:\mathrm{x}\mathrm{x}\:\to\:\mathrm{R}\mathrm{x})\:\to\:(\mathrm{F}\mathrm{x}\mathrm{x}\mathrm{y}\:\to\:\:\mathrm{R}\mathrm{y})$$

$$\:\mathrm{C}\mathrm{O}\mathrm{N}.\:\mathrm{E}\mathrm{X}\mathrm{P}.\:(\mathrm{P}\mathrm{x}\mathrm{y}\:\wedge\:\:\mathrm{x}@\mathrm{z})\:\to\:\:(\exists\:\mathrm{w}(\mathrm{y}@_\mathrm{w})\:\to\:\:\exists\:\mathrm{w}(\mathrm{y}@_\mathrm{w}\:\wedge\:\:\mathrm{P}\mathrm{z}\mathrm{w}\left)\right)$$

$$\:\mathrm{W}-\mathrm{D}\mathrm{E}\mathrm{L}.\:\exists\:\mathrm{y}\left(\mathrm{P}\mathrm{P}\mathrm{y}\mathrm{x}\right)\:\to\:\:(\mathrm{x}@_\mathrm{w}\mathrm{z}\:\to\:\:\exists\:\mathrm{y}(\mathrm{P}\mathrm{P}\mathrm{y}\mathrm{x}\:\wedge\:\:\mathrm{y}@_\mathrm{w}\mathrm{z}\left)\right)$$

$$\:\mathrm{L}\mathrm{O}\mathrm{C}.\:\mathrm{S}\mathrm{U}\mathrm{P}.\:\neg\:\mathrm{x}@\mathrm{y}\:\to\:\:(\mathrm{P}\mathrm{z}\mathrm{x}\:\to\:\:\neg\:\mathrm{z}@\mathrm{y})\:\vee\:\:\exists\:\mathrm{w}\exists\:\mathrm{v}(\mathrm{P}\mathrm{w}\mathrm{x}\:\wedge\:\:\mathrm{D}\mathrm{v}\mathrm{y}\:\wedge\:\:\mathrm{w}@_\mathrm{w}\mathrm{v})$$

I believe the adoption of these axioms is sufficient to ensure that the locational relations in the present theory behave in ways that are reasonably similar to those in which we would intuitively expect them to behave, while also avoiding the entailment of any principle that we would rather leave out of the theory. It is likely that the list of axioms above could be shortened by adopting different principles that imply multiple of ours, but in the presentation of the theory I chose to forsake conciseness in order to hopefully increase the clarity of explanation and the ease of comparison with other theories. Of course, the list above could also be expanded by adding any principles which, though not currently entailed, are considered plausible enough to figure in a theory of location.

## Conclusion

In the first section of this paper, we have characterized two ways of being neutral: neutrality with respect to principles, and neutrality with respect to counterexamples. To illustrate how a theory that is neutral with respect to a certain principle may not be able to account for counterexamples that have been given to the principle in question, some counterexamples to Exactness and Functionality have been considered, including point-like objects in gunky space, omnipresent objects in junky space, enduring objects and immanent universals. It has been shown that, even though many one-primitive theories in the literature are neutral with respect to Exactness and Functionality, none is able to account for all the counterexamples we have considered. More specifically, no one-primitive theory can account for inexact objects, nested multilocation (present in some cases involving immanent universals) and failures of Additivity (present in both cases involving enduring objects and cases involving immanent universals). This is surprising, since the counterexamples in question are relatively commonplace in discussions of location.

The second section of this paper has been devoted to the presentation of a one-primitive theory designed to account for all the aforementioned cases. It is also shown how popular locational principles like Expansivity and Delegation can be modified to be compatible with the negations of Functionality and Exactness. It must be noted that the theory I have presented is not supposed to be perfectly neutral; in fact, some of the choices I have made in its axiomatization, like the assumption of Conditional Reflexivity, might be seen as controversial. In addition, it is not hard to devise new scenarios that the theory cannot account for. For example, my definition of pervasive location allows an omnipresent object to pervade every region of a junky space without being exactly located at any of them, but only as long as the omnipresent object has some part that is exactly located. Moreover, the theory can give unwanted results in some cases involving multiply entirely located inexact objects.

Despite its limitations, I believe the theory I have proposed fills a relevant gap in the literature, as it can account for a set of popular counterexamples that no other one-primitive theory can account for. It also serves to highlight two key points that have been mostly overlooked. The first is that the choice between self-located and non-located regions is philosophically relevant, because it has effects on the rest of the theory that influence its ability to account for certain cases. The second is that it is possible to build a theory of location that has exact location as its only primitive and yet does not entail Exactness and related principles.

## Footnotes

1 To be clear, the notion I mean to investigate is just neutrality *simpliciter*, not *topic*-neutrality; it would be interesting to consider to what extent various theories of location are topic-neutral, but that is not the purpose of this paper. The topic-neutrality of a given theory would plausibly have to do with what sorts of entities are allowed to be occupants or regions in the theory. For an early discussion of this issue, see Simons (2004).

2 Since theories of location are almost always built deductively, by listing axioms and definitions, it may not always be immediately clear what classes of models can be constructed in a given theory. Moreover, it may not always be clear whether a certain model adequately represents a given scenario. But in the rest of the paper I will ignore these complications and assume that it is at least in principle always possible to determine whether a well-formulated counterexample corresponds to a given model or not.

3 Here and in the rest of this paper I have omitted the initial string of universal quantifiers to improve readability; all the formulas should be taken to be implicitly prefixed with universal quantifiers binding all the variables that are not explicitly bounded.

4 This case can be taken as belonging to a more general class of scenarios in which all the available regions are somehow larger than a given object, so that no region is an exact “fit” for the object. Another example would be Kleinschmidt’s (2016) “Almond in the Void” scenario, where the Void is an extended simple region and the Almond an occupant smaller in size than the Void.

5 Notice that this is not the only valid interpretation of endurance: it could be characterized mereologically, in terms of temporal parts, rather than locationally (see Gilmore, 2006, 2008).

6 This general version of Additivity is stated in plural logic and makes use of two predicates that take plural variables as arguments: “x : xx” (read as “x is one of the xx”) and “Fxxz” (read as “z is a fusion of the xx). A weaker, binary version of Additivity could be easily stated in classical first-order logic, by using a three-place predicate of binary fusion (“w = y+z”, read as “w is a fusion of y and z”). Binary Additivity, however, cannot fully account for objects with infinitely many exact locations, because it does not entail that pluralities of infinitely many regions have fusions.

7 An alternative view holds that immanent universals are exactly located only at the fusion of all the locations of their instances, which means that they are not multilocated (Effingham, 2015); this view, however, presents the disadvantage of being unable to reduce the instantiation of a certain universal to a matter of co-location with that universal.

8 Unless explicitly ruled out, there could be a region that is both simple and extended; this is the case in the Almond in the Void example from Kleinschmidt (2016). If a simple object was located at an extended simple region, it should intuitively count as an extended simple, but it would not count as a spanner.

9 No Spanners, as the name indicates, is the principle stating that there are no spanners.

10 If we define a locational relation as holding between an occupant and a region, then the proxy relation is not locational, given that it holds between two occupants. It could be argued that, if the only available intuitive characterizations of the proxy relation have to be given in locational terms (e.g. x is a proxy for y whenever y is always indirectly located in the same regions in which x is directly located), then the proxy relation should still be taken to count as a locational primitive, even though it is not strictly speaking a locational relation. Of course, this worry would be immediately dissipated if there was a way to characterize the proxy relation that does not depend on locational notions. In general, the case for the adoption of the proxy relation as a primitive would be much stronger if there was some independent motivation or use case for it outside of the theory of location.

11 I am grateful to an anonymous referee for bringing up this case.

12 Strong Supplementation is the principle stating that for any x and y, if x is not a part of y, then there is a part of x that is disjoint from y. Together with Antisymmetry, it entails the Extensionality of proper parthood, which is why this theory is known as Extensional Mereology (Cotnoir & Varzi, 2021: 111).

13 This principle appears as (7.26) in Casati and Varzi (1999: 124).

14 This principle can also be found in Eagle (2019: 165–166), and as (L.8’) in Casati and Varzi (1999: 124).

15 This principle first appears in Casati and Varzi (1999). Parsons (2007) calls it Weak Expansivity.

16 Parsons (2007) just calls it Expansivity.

17 Eagle (2019) just calls this Delegation.

18 This principle is similar to one that Correia (2022: 567) calls Locational Strong Supplementation; one of the main differences is that Correia’s principle has entire location, instead of exact location, in the antecedent.

## References

[CR1] Armstrong, D. (1978). *Nominalism and Realism: Universals and Scientific Realism* (Vol. vol. 1). Cambridge University Press.

[CR2] Bigelow, J. (1988). *The Reality of Numbers: A Physicalist’s Philosophy of Mathematics*. Oxford University Press.

[CR3] Calosi, C. (2023). Extended Simples, Unextended Complexes. *Journal of Philosophical Logic*, *52*(2), 643–668.

[CR4] Casati, R., & Varzi, A. (1999). *Parts and Places: The Structure of Spatial Representation*. MIT Press.

[CR5] Correia, F. (2022). A general theory of location based on the notion of entire location. *Journal of Philosophical Logic*, *51*, 555–582.

[CR6] Correia, F. (2025). Location by proxy. *Journal of Philosophical Logic*, *54*, 143–167.

[CR7] Costa, D., & Calosi, C. (2022). The multi-location trilemma. *Erkenntnis*, *87*(3), 1063–1079.

[CR8] Cotnoir, A. J., & Varzi, A. (2021). *Mereology*. Oxford University Press.

[CR9] Eagle, A. (2010). Perdurance and Location. In D. Zimmerman (Ed.), *Oxford Studies in Metaphysics* (Vol. 5, pp. 53–94). Oxford University Press.

[CR10] Eagle, A. (2016). Multiple location defended. *Philosophical Studies*, *173*(8), 2215–2231.

[CR11] Eagle, A. (2019). Weak location. *Dialectica,**3*(1–2), 149–181.

[CR12] Effingham, N. (2015). The location of properties. *Nous*, *49*, 25–44.

[CR13] Gilmore, C. (2006). Where in the relativistic world are we? *Philosophical Perspectives*, *20*, 199–236.

[CR14] Gilmore, C. (2007). Time travel, coinciding objects, and persistence. In D. Zimmerman (Ed.), *Oxford Studies in Metaphysics* (Vol. 3, pp. 177–198). Oxford University Press.

[CR15] Gilmore, C. (2008). Persistence and location in relativistic spacetime. *Philosophy Compass*, *3*, 1224–1254.

[CR16] Hudson, H. (2005). *The Metaphysics of Hyperspace*. Oxford University Press.

[CR17] Kleinschmidt, S. (2016). Placement permissivism and logics of location. *Philosophical Perspectives*, *25*, 253–276.

[CR18] Leonard, M. (2014). Locating gunky water and wine. *Ratio*, *27*(3), 306–315.

[CR19] Lewis, D. (1976). The paradoxes of time travel. *American Philosophical Quarterly*, *13*, 145–152.

[CR20] Lewis, D. (1986). *On the Plurality of Worlds*. Blackwell.

[CR21] Loss, R. (2021). Somewhere together: Location, parsimony and multilocation. *Erkenntnis*, *88*(2), 675–691.

[CR22] Parsons, J. (2007). Theories of location. In D. Zimmermann (Ed.), *Oxford Studies in Metaphysics* (Vol. 3, pp. 201–232). Oxford University Press.

[CR23] Pickup, M. (2016). Unextended complexes. *Thought: A Journal of Philosophy*, *5*(4), 257–264.

[CR24] Saucedo, R. (2011). Parthood and location. In K. Bennett, & D. Zimmermann (Eds.), *Oxford studies in metaphysics* (Vol. 6, pp. 225–284). Oxford University Press.

[CR25] Simons, P. (2004). Location. *Dialectica,**58*(3), 341–347.

[CR26] Uzquiano, G. (2011). Mereological harmony. In K. Bennett, & D. Zimmermann (Eds.), *Oxford studies in metaphysics* (Vol. 6, pp. 199–224). Oxford University Press.

